# Dreams and visions at the end of life: Lessons from a patient encounter

**DOI:** 10.1017/S1478951525100953

**Published:** 2025-10-23

**Authors:** Intissar Belrhali, Stephane Ruck, Hind Mrabti, Hassane Errihani

**Affiliations:** 1 Department of Oncology, National Institute of Oncology, Rabat, Morocco; 2 Department of Medical Oncology, Emile Durkheim Hospital Center, Épinal, France

I still remember a patient I accompanied during my fellowship in Épinal, France. She was in the final stage of her illness, receiving palliative care in her last weeks of life. A devoted Catholic, she carried her faith with remarkable strength despite the frailty of her body.

In those last days, she frequently shared with us her dreams and visions. She described, with striking clarity, seeing her late husband smiling at her bedside, hearing hymns from her childhood, and walking through a luminous garden filled with peace. Sometimes these moments came in the quiet of the night, sometimes while she was awake, resting.

What was most striking was her serenity. These experiences did not bring fear but comfort. For her, they were signs of reunion, of continuity, of God’s presence waiting for her. They gave her meaning in a moment when medicine could do little more.

Her family, however, did not always know how to respond. They moved between doubt and belief – wondering if these were signs of confusion, or perhaps a grace preparing her for what was to come. Their ambivalence reflected both love and uncertainty: they wanted to protect her, yet also to trust her words.

As clinicians, we too hesitated at first. Should we interpret these experiences as hallucinations? Should we intervene medically to suppress them? Or should we listen and respect them for what they were to her?

Over time, it became clear that what she needed most was not explanation but recognition. She wanted her words to be heard, not dismissed. Alongside the care we provided for her physical suffering, the most important support we offered was presence – listening without judgment, and reassuring her family that such experiences are common at the end of life and often bring peace rather than fear.

Gradually, her family began to see her dreams differently. What had seemed a cause for worry became a source of comfort. They recognized these visions not as confusion but as bridges, helping her to let go.

For me, this encounter was transformative. It reminded me that palliative care is not only about managing pain or easing breathlessness, but also about honoring experiences that reach beyond medical categories. For this patient, her dreams were not pathology but prayer; not confusion but consolation. By respecting them, we respected her identity, her faith, and her dignity.

Since then, I have learned that such experiences are not rare. Many patients in their last days describe vivid dreams and visions of loved ones, journeys, or light. They are often sources of peace and meaning, not distress. These moments may not fit into the language of medicine, but they are part of the reality of dying.

This patient taught me a lesson that continues to shape my practice: that as palliative care providers, our role is not to resolve every mystery, but to recognize its value. To accompany someone at the end of life means listening not only to their pain, but also to their visions, their faith, and their inner world.

As I look back, I remain grateful to her. She showed me that dying is not only a medical process, but also a deeply human and spiritual journey. Her dreams helped her, and her family, face death with less fear and more meaning. They helped me see that in the silence of the end of life, there is often more light, comfort, and hope than we dare to imagine.

